# Nox2 Deficiency Prevents Hypertension-Induced Vascular Dysfunction and Hypertrophy in Cerebral Arterioles

**DOI:** 10.1155/2013/793630

**Published:** 2013-03-14

**Authors:** Siu-Lung Chan, Gary L. Baumbach

**Affiliations:** ^1^Department of Pathology, University of Iowa Carver College of Medicine, 5231D RCP, 200 Hawkins Drive, Iowa City, IA 52242, USA; ^2^Department of Neurological Sciences, University of Vermont, 149 Beaumont Avenue, HSRF 416, Burlington, VT 05405, USA

## Abstract

Oxidative stress is involved in many hypertension-related vascular diseases in the brain, including stroke and dementia. Thus, we examined the role of genetic deficiency of NADPH oxidase subunit Nox2 in the function and structure of cerebral arterioles during hypertension. Arterial pressure was increased in right-sided cerebral arterioles with transverse aortic banding for 4 weeks in 8-week-old wild-type (WT) and Nox2-deficient (-/y) mice. Mice were given N^G^-nitro-L-arginine methyl ester (L-NAME, 10 mg/kg) or vehicle to drink. We measured the reactivity in cerebral arterioles through open cranial window in anesthetized mice and wall cross-sectional area and superoxide levels *ex vivo*. Aortic constriction increased systolic and pulse pressures in right-sided carotid arteries in all groups of mice. Ethidium fluorescence showed increased superoxide in right-sided cerebral arterioles in WT, but not in Nox2-/y mice. Dilation to acetylcholine, but not sodium nitroprusside, was reduced, and cross-sectional areas were increased in the right-sided arterioles in WT, but were unchanged in Nox2-/y mice. L-NAME reduced dilation to acetylcholine but did not result in hypertrophy in right-sided arterioles of Nox2-/y  mice. In conclusion, hypertension-induced superoxide production derived from Nox2-containing NADPH oxidase promotes hypertrophy and causes endothelial dysfunction in cerebral arterioles, possibly involving interaction with nitric oxide.

## 1. Introduction

Chronic hypertension is a major risk factor of vascular disorders. Profound vascular functional and structural changes occur in many disease states, and emerging evidence suggests that oxidative stress has a major role in mediating these changes [1]. For example, increased oxidative stress has been shown in hypertension-induced vascular diseases, stroke and subcortical vascular dementia [2–4]. Moreover, oxidative stress from various sources has been implicated in endothelial dysfunction and structural remodeling in the cerebral vasculature [5, 6]. 

Although there are many sources of reactive oxygen species (ROS), the primary source of superoxide production in the vascular wall is thought to be NADPH oxidase [7]. NADPH oxidase consists of a membrane-bound *b*-type cytochrome composed of 91 and 22 kDa subunits (referred to as gp91^phox^ (also known as Nox2) and p22^phox^, resp.), and three cytosolic proteins (p47^phox^, p67^phox^, and p21^rac^) [7]. While functional forms of NADPH oxidase have been demonstrated throughout the vasculature, there are subtle, but important, structural differences with respect to its subunits depending on vessel size and region. For example, it appears that Nox2 may play a role in NADPH oxidase activity in vascular muscle of small resistance arteries, whereas homologues of Nox2, such as Nox1 and Nox4, may be more important in large cerebral arteries [8, 9]. Furthermore, a number of studies showed that angiotensin II-induced impairment of endothelial function and reduced cerebral blood flow are restored in Nox2-deficient (-/y) mice [10, 11]. These findings highlight the importance of Nox2-containing NADPH oxidase in the pathology of hypertension in the cerebral circulation.

Cerebral resistance arteries and arterioles play critical roles in controlling local cerebral blood flow [12]. It is, therefore, important to understand the mechanism for functional and structural changes during chronic hypertension in these small brain vessels. In an effort to determine the source of ROS in cerebral arterioles during hypertension, the first goal of this study was to examine the hypothesis that Nox2-derived ROS results in vascular dysfunction and hypertrophy in cerebral arterioles during hypertension. We used a transverse aortic banding procedure to increase cerebral vascular pressure and oxidative stress in the right side of the brain [13, 14]. An advantage of this model is that the left cerebral hemisphere remains normotensive relative to the right side, and thus vessels in the left hemisphere can be used as normotensive controls. 

Nitric oxide (NO) is a major mediator of endothelium-dependent dilation and inhibits mitogenesis and proliferation of vascular smooth muscle cells [15]. NO readily reacts with superoxide; thus, the local concentration of superoxide is an important determinant of the biological availability of NO [16]. A previous study has demonstrated the relevance of the NO-dependent pathway in endothelial dysfunction and hypertrophy in cerebral vasculature [17]. Thus, our second goal was to examine the hypothesis that the NO-dependent pathway plays a role in Nox2-derived ROS-induced dysfunction and hypertrophy of cerebral arterioles during chronic hypertension.

## 2. Methods

### 2.1. Animals

Nox2-/y and wild-type (WT) mice were purchased from Jackson Laboratory (Bar Harbor, ME, USA). Animals were housed in pathogen-free facility at 24°C, exposed to 12 hours of light (lights on at 06:00, off at 18:00) and allowed free access of food and fluid. All animals were studied at 13 to 15 weeks of age. Procedures followed in this study were approved by the Institutional Animal Care and Use Committee of the University of Iowa.

### 2.2. Transverse Aortic Banding

Increased pressure in the proximal aorta in all animals was induced by means of thoracic aortic banding using the method described previously [18]. Briefly, mice were anesthetized with ketamine (100 mg/kg, i.p.) plus xylazine (5 mg/kg, i.p.), intubated with 20-gauge tubing and ventilated (Harvard Apparatus Rodent Ventilator, model 687) at 100 breaths per minute (0.1 mL tidal volume). Thoracotomy was created at the second intercostal space. The transverse aortic arch was ligated (7–0 Prolene) between the innominate and left common carotid arteries with an overlying 27-gauge needle, and then the needle was removed, leaving a discrete region of stenosis. The 24 h and 1-week survival rate were each higher than 90%.

### 2.3. L-NAME Treatments

L-NAME (10 mg/kg/day, 4 weeks) was given in drinking water to WT (*n* = 16) and Nox2-/y (*n* = 16) mice. This dose regimen has been shown to induce hypertrophy in cerebral arterioles [17]. The treatment was started the same day after aortic banding. We replaced freshly prepared L-NAME solution everyday or every other day. We adjusted the concentration of L-NAME every time based on the volume an individual mouse drank.

### 2.4. Determination of Blood Pressures in Conscious Animals

Systemic arterial blood pressures were measured in 6 mice from each group using an automated tail-cuff device (Visitech Systems BP-2000, Apex, NC, USA). Mice were placed in specifically designed mouse holders that allow measurement of systolic blood pressure under resting conditions. Mice were trained for 5 days, and then blood pressure was measured at days 0 (baseline), 7, 14, 21, and 28 of treatment. Each day, 30 measurements were made and averaged for each mouse.

### 2.5. Determination of Cerebral Arteriolar Diameter and Structure

Four weeks after aortic banding, we measured diameter in first-order arterioles on the surfaces of the right and left cerebral hemispheres through an open skull preparation as described in detail previously (*n* = 8 in each group) [19, 20]. Cerebral arterioles were monitored through a microscope connected to a closed-circuit video system with a final magnification of ×356. Arteriolar diameter was measured from digitized images of arterioles using NIH Image version 1.62 (National Institute of Health, USA). About 30 minutes after completion of craniotomy, cerebral arterioles were exposed to acetylcholine (ACh, 10^−5^ M) dissolved in artificial cerebral spinal fluid (CSF) for 5 min. Arteriolar diameters were measured and drug was washed by CSF for 5 min. The procedure was repeated with sodium nitroprusside (SNP, 10^−7^ M). In addition, systemic arterial pressure was measured continuously via catheters inserted into the right and left common carotid arteries.

 To determine whether increases in arterial pressure that result from transverse aortic banding are limited to the right side of the brain, pressure was measured in right- and left-sided first-order cerebral arterioles in a separate group of anesthetized WT mice (*n* = 6) using a servo-null system as described in detail previously [19, 20]. The mice had undergone the transverse aortic banding procedure 4 weeks before measuring arteriolar pressure. Systolic (SP), diastolic (DP), mean (MP), and pulse (PP) pressures were significantly higher (*P* < 0.05) in right-sided (62 ± 6, 35 ± 2, 44 ± 3, and 28 ± 4 mmHg; SP, DP, MP, and PP, resp.) than in left-sided cerebral arterioles (39 ± 3, 28 ± 2, 32 ± 2, and 10 ± 1 mmHg; SP, DP, MP, and PP, resp.). Furthermore, the levels of SP, DP, MP, and PP in left-sided arterioles in aortic banded mice were similar to those we observed previously in normotensive WT mice [5, 6].

In another set of animals (*n* = 8 in each group), structural characteristics were studied using the same open cranial window technique. After the baseline diameters were measured, arterioles then were suffused with CSF-containing EDTA (67 mM), which produces maximal dilation of cerebral arterioles [21]. Arterioles were fixed at physiological pressure *in vivo* by suffusion of vessels with glutaraldehyde fixative (2.25% glutaraldehyde in 0.1 M cacodylate buffer) while maintaining cerebral arteriolar pressure at baseline levels. After the anesthetized animal was euthanized using overdose sodium pentobarbital, cerebral arteriolar segments and carotid arteries were removed, processed, and embedded in Spurr's low-viscosity resin while maintaining cross-sectional orientation. Cross-sectional area (CSA) of the vessel wall was determined histologically using a method described previously [21].

### 2.6. Determination of Superoxide in Cerebral Arterioles

In another set of animals (*n* = 8 in each group), superoxide levels were evaluated *in vitro* in 6–8 *μ*m thick frozen sections of unfixed right- and left-sided cerebral arterioles using hydroethidine-based (2 *μ*M hydroethidine) confocal microscopy as described previously [22]. Laser settings were identical for the acquisition of all images, and vessels from WT and Nox2-/y mice were processed and imaged in parallel. Relative increases in ethidium fluorescence were determined and normalized to the cross-sectional area of the vessel wall.

### 2.7. Drugs

ACh, SNP, and L-NAME were purchased from Sigma (St. Louis, MO, USA). ACh and SNP were dissolved in artificial CSF. L-NAME was dissolved in distilled water.

### 2.8. Statistical Analysis

Analysis of variance was used to compare blood pressure, cerebral arteriolar diameters, cross-sectional areas, and superoxide levels of the vessel wall. Probability values were calculated using Graph Pad Prism 5 (Graph Pad Software, Inc., San Diego, CA, USA). Values were presented in mean ± SEM and were considered different when *P* < 0.05 using post hoc Bonferroni test.

## 3. Results

### 3.1. Nox2 Deficiency Inhibits Hypertension-Induced Superoxide Production in Cerebral Arteriole

**Figure 1:**
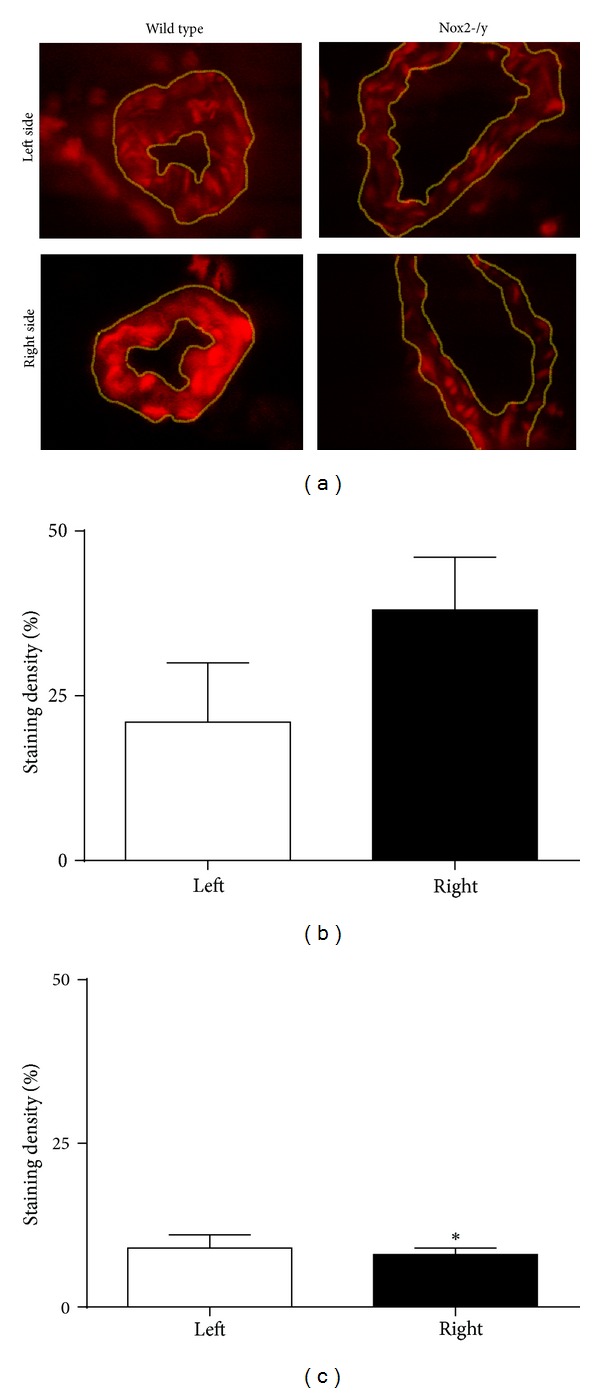
Representative micrographs of superoxide levels determined by hydroethidium fluorescence of WT left-sided, WT right-sided, Nox2-/y left-sided, and Nox2-/y right-sided cerebral arterioles (highlighted in yellow) (a). Graphs showing relative staining density of WT (b) and Nox2-/y mice (c) (*n* = 8).  *P < 0.05 versus WT right-sided cerebral arterioles.

To determine if Nox2-containing NADPH oxidase is responsible for hypertension-induced superoxide production, levels of superoxide were determined in cerebral arterioles from WT and Nox2-/y mice by ethidium fluorescence. Representative micrographs show that fluorescence of ethidium was higher in right- than left-sided cerebral arterioles in WT mice (Figure 1(a), left panel). In contrast, fluorescence in right-sided arterioles did not appear to be increased in Nox2-deficient mice (Figure 1(a), right panel), and fluorescence in left-sided arterioles appeared to be lower in Nox2-deficient mice than in WT mice. Semiquantification of ethidium signal confirmed that levels of fluorescence were higher in right- than left-sided cerebral arterioles in WT mice (Figure 1(b)) and similar in right-and left-sided arterioles in Nox2-/y mice (Figure 1(c)). These findings suggest that Nox2 is the major source of hypertension-induced superoxide in cerebral arterioles.

### 3.2. Nox2 Deficiency Did Not Alter Blood Pressure

**Figure 2:**
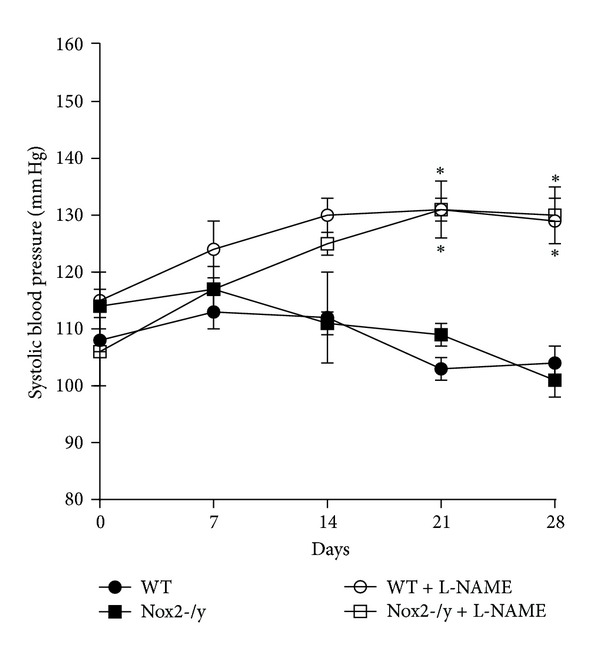
Graph showing systolic blood pressures measured by tail-cuff method. Results were the averages of thirty measurements made on days 0, 7, 14, and 28 of 6 individual mice.  *P < 0.05 versus corresponding control treatment group.

SDs measured under conscious conditions by a tail-cuff method prior to L-NAME treatment were similar in WT and Nox2-/y mice (Figure 2). Aortic banding did not alter conscious SP in any of the animals. Blood pressure of WT and Nox2-/y mice was increased in the last two weeks of L-NAME treatment. Response to L-NAME was similar in both strains.

Pressures were measured in right and left carotid arteries in anesthetized mice to confirm that transverse aortic banding was successful. SP and PP, but not DP and MP, were significantly increased by similar levels in right- compared to left-sided carotids in untreated WT and untreated Nox2-/y mice (Table 1).

### 3.3. Nox2 Deficiency Prevents Hypertension-Induced Endothelial Dysfunction in Cerebral Arterioles

**Figure 3:**
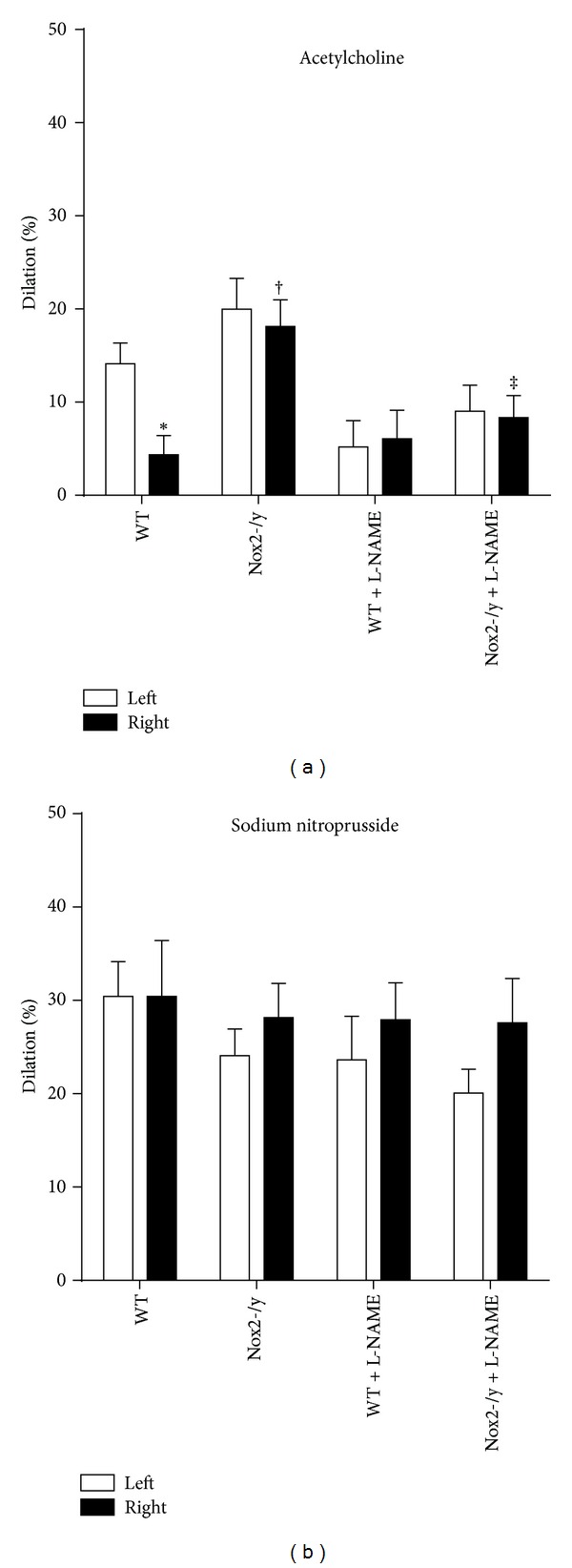
Graph showing endothelium-dependent and -independent vasodilation in cerebral arterioles. Acetylcholine (Ach, 10^−5^ mol/L) (a) or sodium nitroprusside (SNP, 10^−7^ mol/L) (b) was suffused in artificial CSF for 5 min in 8 mice.  *P < 0.05 versus left-sided WT group; ^†^ *P* < 0.05 versus right-sided WT group; and ^‡^ *P* < 0.05 versus right-sided Nox2-/y group.

To test whether endothelial dysfunction induced by hypertension is Nox2-dependent, dilator responses to ACh and SNP were studied. Dilator response to ACh was significantly decreased in right-sided cerebral arterioles relative to left-sided arterioles in untreated WT mice, suggesting an endothelial dysfunction on the hypertensive side (Figure 3(a)). Response to SNP was similar in both sides of WT mice, indicating that aortic banding did not affect smooth muscle contractility (Figure 3(b)). In untreated Nox2-/y mice, responses to Ach were restored in right-sided cerebral arterioles comparable to that of the left side, suggesting normal endothelial function. Treatment with L-NAME reduced dilator responses in left-sided cerebral arterioles to ACh, but not SNP, in both WT and Nox2-/y mice. Moreover, L-NAME blunted dilator responses to ACh in right-sided arterioles in Nox2-/y mice.

### 3.4. Nox2 Deficiency Prevents Hypertension-Induced Hypertrophy in Cerebral Arterioles

**Figure 4:**
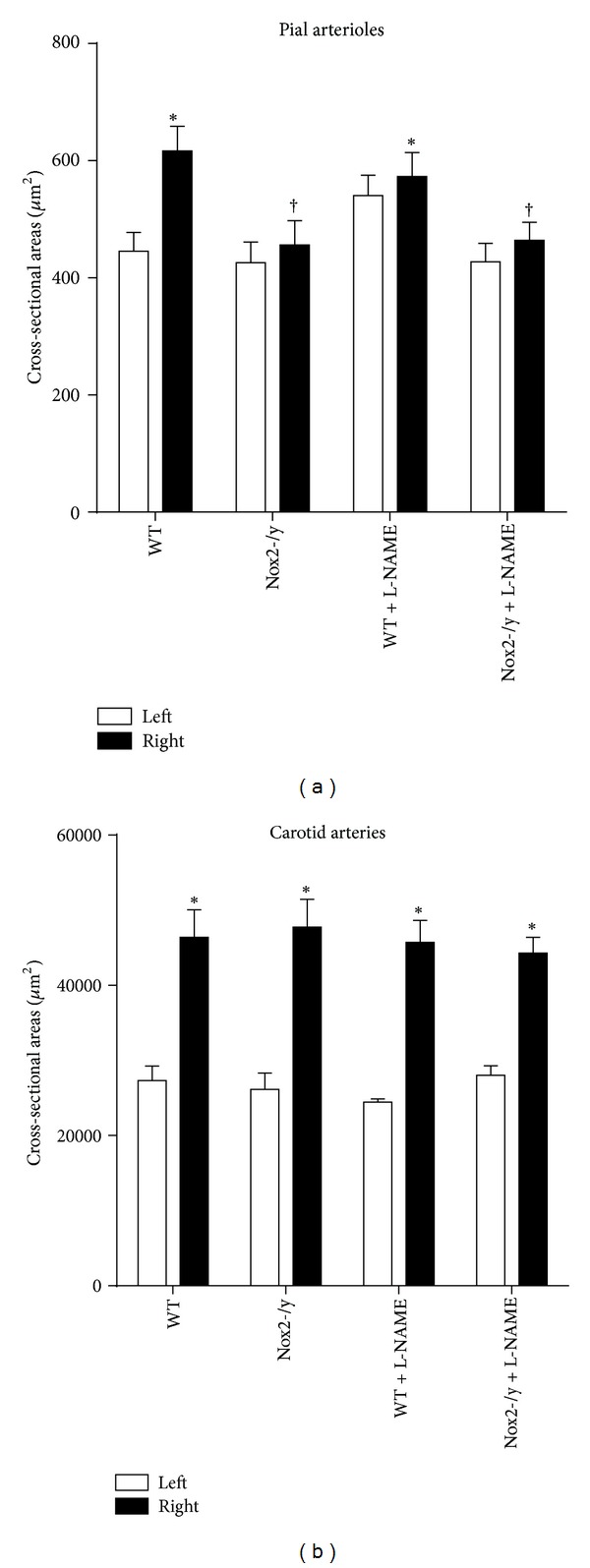
Graph showing cross-sectional areas of maximally dilated cerebral arterioles (a) and carotid arteries (*n* = 8) (b). *P < 0.05 versus left-sided WT group; ^†^ *P* < 0.05 versus right-sided WT group.

To determine whether Nox2 contributes to hypertension-induced hypertrophy in cerebral arterioles, we measured CSA of the arteriolar wall. CSA of the arteriolar wall was greater in right-, than in left-sided, cerebral arterioles in untreated WT mice, but not in untreated Nox2-/y mice (Figure 4(a)). Treatment with L-NAME increased CSA in left-sided, but not right-sided cerebral arterioles, in WT mice. In contrast, L-NAME did not produce hypertrophy in either right- or left-sided arterioles in Nox2-/y mice, which suggests an important role for Nox2-dependent production of ROS in the development of hypertension-induced cerebral arteriolar hypertrophy. In contrast to cerebral arterioles, Nox2 deficiency did not prevent increases in CSA of the vessel wall in carotid arteries (Figure 4(b)), suggesting that Nox2 does not contribute to hypertension-induced hypertrophy in larger conduit arteries.

## 4. Discussion

Chronic hypertension has profound impacts on the vasculature and is a known risk factor for stroke and dementia. It is important to understand the mechanism of vascular changes during chronic hypertension, particularly in smaller resistance arterioles because they provide substantial vascular resistance and are important in controlling local blood flow [12]. In this study, we used a transverse aortic banding model to increase blood pressure to the right, but not the left, side of the brain to study mechanisms of vascular dysfunction and structural remodeling in chronic hypertension. There are several important findings. First, superoxide levels were increased in the hypertensive side of the brain in WT, but not in Nox2-/y mice. This result suggests that Nox2-containing NADPH oxidase is the major source of superoxide in cerebral arterioles during hypertension. Second, deficiency of Nox2 prevented hypertension-induced impairment of endothelium-dependent dilatation in cerebral arterioles. Moreover, L-NAME treatment eliminated the normalized endothelial function in hypertensive cerebral arterioles of Nox2-/y mice, suggesting an NO-dependent mechanism. Third, hypertension caused hypertrophy in cerebral arterioles from WT mice, but not in Nox2-/y mice. This suggests that ROS derived from Nox2-containing NADPH oxidase play a key role in hypertension-induced hypertrophy in cerebral arterioles. Interestingly, Nox2 deficiency did not prevent hypertrophy in carotid artery. Taken together, this study provides *in vivo* evidence that chronic hypertension induces cerebral arteriolar dysfunction and hypertrophy via increased production of ROS derived from Nox2-containing NADPH oxidase. 

We used ethidium fluorescence to examine the effects of Nox2 deficiency on hypertension-induced production of superoxide in cerebral arterioles. Being aware of potential problems with this method, matched pairs of hypertensive (right-sided) and normotensive (left-sided) cerebral arterioles from each mouse were examined in parallel using the same reagents and laser settings. In addition, we have shown previously that incubation with PEG-SOD, a scavenger of superoxide, abolishes ethidium fluorescence in aorta of mice that overexpresses human renin and human angiotensinogen [23].

Chronic hypertension is well known to increase vascular production of ROS. Using a model of abdominal aortic banding, it was shown previously that superoxide levels are elevated in noncerebral vessels [13, 14]. In the present study, transverse aortic banding was used to increase pressure in right-sided (hypertensive) cerebral arterioles relative to left-sided (normotensive) cerebral arterioles. We found in WT mice that the production of superoxide was elevated in right-sided cerebral arterioles relative to left-sided arterioles, whereas in Nox2-/y mice levels of superoxide were essentially the same in right- and left-sided arterioles. This finding indicates that Nox2-containing NADPH is the major source of increased production of ROS in cerebral arterioles during hypertension.

It is still debatable as to whether Nox2-derived ROS play a role in the development of hypertension. For example, the pressor response to angiotensin II was found to be reduced in Nox2-/y mice in one study [24] and unaffected in another [25]. The finding in this study that L-NAME increased systemic blood pressure in Nox2-/y mice to levels similar to those found in WT mice supports the idea that Nox2-derived ROS do not contribute significantly to hypertension. 

Endothelial dysfunction caused by hypertension in the cerebral circulation has been previously demonstrated in various animal models [10, 26]. To our knowledge, this is the first study to examine endothelium-dependent function in cerebral arterioles using the transverse aortic banding model in mice. One of the advantages of this model is that the contralateral side can be used as a normotensive control. Previous studies found disparate effects of hypertension induced by aortic banding on endothelium-dependent function in aorta or coronary arteries [13, 27], probably due to the differences in the vascular beds studied and the location of the band. In this study, our finding that transverse aortic banding impairs endothelium-dependent dilatation in right-sided (hypertensive) cerebral arterioles in WT mice, but not in Nox2-/y mice, suggests that superoxide derived from Nox2-containing NADPH oxidase may play an important role in altered endothelium-dependent function in cerebral arterioles during hypertension. 

Hypertension-induced impairment of endothelium-dependent dilatation is thought to result from reduced availability of NO due either to its destruction by NADPH-derived superoxide or to an uncoupling of eNOS, which results in the production of superoxide instead of NO. Our finding that Nox2 deficiency protected against impairment in cerebral arteriolar dilatation during hypertension produced with transverse aortic banding, but not L-NAME, supports the concept that hypertension impairs endothelial-dependent dilatation of cerebral arterioles through the destruction of NO by NADPH-derived superoxide, and not by uncoupling of eNOS.

Cerebral vascular hypertrophy is a well-known consequence of hypertension [17, 19, 21]. A role for superoxide in the development of vascular hypertrophy is implicated by the finding that mice deficient in copper-zinc superoxide dismutase develop cerebral arteriolar hypertrophy while remaining normotensive [6]. Additional support for this concept is provided by the finding in this study that whereas transverse aortic banding resulted in hypertrophy of hypertensive cerebral arterioles in WT mice, hypertensive arterioles did not undergo hypertrophy in Nox2-/y mice.

One mechanism by which superoxide may promote vascular hypertrophy is through the destruction of NO [28]. NO has been shown to inhibit mitogenesis and proliferation of cultured smooth muscle [29], and treatment with L-NAME, as well as deficiency of eNOS, has been shown to induce hypertrophy of cerebral arterioles in mice [17]. Further support for this concept would appear to be provided by our finding in this study that treatment of aortic banded WT mice with L-NAME tended to cause hypertrophy in normotensive (left-sided), as well as hypertensive (right-sided), cerebral arterioles (*P* = 0.063 versus untreated WT mice). However, the possibility that destruction of NO may not be a critical factor in the development of ROS-induced hypertrophy of cerebral arterioles is suggested by our finding that the treatment of Nox2-dieficient mice with L-NAME did not induce hypertrophy in either hypertensive or normotensive cerebral arterioles. Instead, this finding suggests that ROS derived from Nox2-containing NADPH oxidase may play a central role in the development of cerebral arteriolar hypertrophy. 

While we cannot exclude the possibility that ROS downstream of superoxide, such as peroxynitrite or hydrogen peroxide, contribute to the hypertrophic process, we believe that superoxide plays a more important and direct role in causing cerebral vascular hypertrophy. We base this speculation on two observations. First, we showed in this study that L-NAME inhibition, which supposedly limits the interaction of superoxide and NO to form peroxynitrite, does not attenuate the degree of cerebral arteriolar hypertrophy induced by transverse aortic banding in WT mice. Second, we showed in a previous study that the deficiency of copper-zinc superoxide dismutase, which leads to reduced conversion of superoxide to hydrogen peroxide, nevertheless causes hypertrophy in cerebral arterioles [6]. 

## 5. Conclusion

The present study demonstrated that ROS derived from Nox2-containing NADPH oxidase are critical in hypertension-mediated cerebral arteriolar vascular dysfunction and hypertrophy. This may lead to reduction of dilator capacity and the ability to control local cerebral blood flow during hypertension. 

## Figures and Tables

**Table 1:** Summary of blood pressures (measured by carotid catheters in anesthetized mice) and arterial blood gases in WT and Nox2-/y mice.

Parameters	WT		Nox2-/y		WT + L-NAME		Nox2-/y + L-NAME	
	Left	Right	Left	Right	Left	Right	Left	Right
Systemic arterial pressure (mm Hg)								
Systolic	70 ± 3	90 ± 4*	73 ± 2	95 ± 2*	67 ± 2	91 ± 4*	65 ± 2	84 ± 2*
Diastolic	55 ± 2	55 ± 2	63 ± 2	64 ± 2	52 ± 3	52 ± 3	52 ± 3	53 ± 3
Mean	62 ± 2	68 ± 3	67 ± 2	75 ± 2	58 ± 3	66 ± 3	57 ± 3	64 ± 2
Pulse	13 ± 2	32 ± 3*	9 ± 1	30 ± 3*	14 ± 2	38 ± 5*	12 ± 2	29 ± 3*
Arterial blood gases								
pH	7.39 ± 0.02		7.41 ± 0.01		7.35 ± 0.03		7.33 ± 0.03	
PCO_2_	28 ± 2		27 ± 1		33 ± 3		33 ± 4	
PO_2_	114 ± 9		113 ± 5		104 ± 6		108 ± 8	
Age (week)	12.9 ± 0.6		13.4 ± 0.2		14.7 ± 0.4		14.3 ± 0.7	
Weight (g)	23.1 ± 0.8		22.1 ± 0.5		22.2 ± 0.8		20.5 ± 1.1	
N	14		17		17		15	

*P < 0.05 versus left-sided cerebral arterioles.
